# Evaluation Efficacy of Ferrous Sulfate Therapy on Headaches of 5-15 Years Old Iron Deficient Children with Migraine

**Published:** 2016-03-15

**Authors:** R Fallah, S Zare Bidoki, M Ordooei

**Affiliations:** 1 **Department of Pediatrics, Children Growth Disorders Research Center, Shahid Sadoughi University of Medical Sciences, Yazd, Iran. **; 2 **Department of Pediatrics, Shahid Sadoughi University of Medical Sciences, Yazd , Iran.**

**Keywords:** Adverse effects, Malignancy, Pediatric, Complications, Vascular access device

## Abstract

**Background:**

Some researches have shown the association between iron deficiency and migraine headache in adults. The aim of present study was to evaluate efficacy of ferrous sulfate treatment on migraine headaches of 5-15 years old migraineur children with iron deficiency.

**Materials and Methods:**

In a quasi- experimental study, monthly frequency, severity, duration and disability of headaches of 5-15 years old migraineur children that prophylactic therapy was indicated in them and had iron deficiency who were referred to Pediatric Neurology Clinic of Shahid Sadoughi University of Medical Sciences, Yazd, Iran between 2013 and 2015 and were treated with 2mg/kg/day topiramate plus 4mg/kg/day of ferrous sulfate for three consecutive months, were evaluated and headache characteristics before and after treatment were compared.

**Results:**

In this study, 98 children with mean age of 9.72±3.19 were evaluated that 31children (31.6%) had iron deficiency. Monthly frequency (22.89±7.18 vs.14.5±4.56, P= 0.02), severity score (8.12± 1.76 vs. 5.03±1.15, P= 0.02) and disability score of headache (38.23±10.7vs. 30.12±7.46, P= 0.03) were more in children with iron deficiency. Iron therapy was effective in decreasing of monthlyfrequency 22.89± 7.18 vs. 10.13±4.51, P = 0.001), severity score (8.12±1.76 vs. 5.11±1.62, P =0.001), duration (2.14±1.23 vs.1.14±1.01, P= 0.001) and disability score of headache (38.23±10.7 vs. 22.87±8.65, P= 0.01).

**Conclusion:**

In children, iron deficiency increased monthly frequency, severity and disability of migraine headache and ferrous sulfate can be used as a safe and effective drug in migraine prophylaxis.

## Introduction

Migraine is one of the most common primary headaches of children and 10.6 % of 5-15 years old children have migraine headache. Migraine prevention therapy should be started if headaches occur more than 3-4 times per month or if headache disrupts daily function and the child has Pediatric Migraine Disability Assessment score (pedMIDAS) of more than 20 (1). Many drugs such as propranolol, cyproheptadine, flunarizine, amitriptyline, topiramate, valproic acid and levatiracetam have been used for prevention of migraine headache in children, but none of them have been approved by Food and Drug Administration. Flunarizine is probably effective, but it is not available in many countries including Islamic Republic of Iran and more studies for the use of preventive drugs in migraine headaches of children should be done (2, 3). Efficacy of topiramate in dosage of 2 mg/kg/day for prevention of pediatric migraine headache was shown in a study in Yazd, Iran (4) .

On the other hand, iron deficiency (ID) is the most frequent micronutrient deficiency that affects at least one third of the population of the world. Anemia is the most common clinical manifestation of iron deficiency, but, other organs and systems may also be affected (5).

Cognitive dysfunction, psychomotor retardation, behavioral problems, pica, breath holding spells, restless leg syndrome and thrombosis can be associated with iron deficiency. Effect of ID on brain and its mechanisms as altered development of hippocampus neurons, impairment of energy metabolism, delayed maturation of myelin, and alterations in synaptic neurotransmitter systems including norepinephrine, dopamine, glutamate, gamma-amino butyric acid, serotonin, monoamine and aldehyde oxidases may be responsible for these neurobehavioral symptoms (5). Iron deficiency is one of the most important public health problems in the Middle Eastern countries with moderate to severe prevalence (6). In a study by Keikhaei et al., in Iran, prevalence of iron deficiency and iron deficiency anemia (IDA) in 6-60 month old children were 43.9% and 29.1%, respectively (7).Two research in Iran on adult patients have investigated association between iron deficiency and migraine headache (8, 9) and based on result of Keivani et al. study in Shahrekord, Iran, iron deficiency was more frequent in 15-45 years old women with migraine headache than women without migraine headache (8) and another study in Shahrekord, Iran showed that ferrous sulfate therapy was effective in reducing of monthly frequency and severity of migraine headache of 15-45 years old migrainer women with and without iron deficiency (9).

The aim of present research was to answer this question that whether or not ferrous sulfate treatment can reduce monthly frequency, severity, duration and disability score of migraine headaches in 5-15 years old migraineur children with iron deficiency.

## Materials and Methods 

In this quasi- experimental study, monthly frequency, severity, duration and disability score of migraine headaches of 5-15 years old migraineur children that prophylactic therapy was indicated in them and were referred to the Pediatric Neurology Clinic of Shahid Sadoughi University of Medical Sciences, Yazd, Iran from April 2013 to 2015 were evaluated and among them, patients with iron deficiency were selected and treated with 2 mg/kg/day of topiramate and 4 mg/kg/day of ferrous sulfate for three months and migraine headache characteristics were compared before and three months after iron therapy. The sample size of 30 children was assessed based on Z formula and a confidence interval of 95% with 80% power to detect any significant difference between two groups with a significance level of 0.05.

Eligible participants included those who aged 5-15 years, had migraine headache (with or without aura) based on second edition of the International Classification of Headache Disorders criteria (10) in clinical evaluation by a pediatric neurologist for at least six months before the study, had not used any migraine preventive therapy , had frequent (one or more headache attack per week) or disabling [ Pediatric Migraine Disability Assessment score (pedMIDAS) more than 20] headaches that prophylactic therapy was indicated in them and they had also iron deficiency.Venous blood sample was obtained from all the children and their hemoglobin level, hematocrit, mean corpuscular volume (MCV), serum ferritin level, serum iron level and total iron-binding capacity (TIBC) were measured in Shahid Sadoughi Hospital laboratory. Anemia was defined as hemoglobin level of less than 11.5g/dl and iron deficiency was defined as serum ferritin level of less than 12 ng/mL in afebrile patient, serum iron level of less than 22μ g/dL or transferrin saturation (a percentage cal¬culated as serum iron concentration /TIBC × 100) of less than 16 percent. The criterion had a sensitivity of 75% and a specificity of 76% (11-13). Exclusion criteria included presence of systemic diseases (renal, cardiac, hepatic, haemostatic, diabetes mellitus, etc) based on clinical and laboratory screening evaluation, headaches other than migraine and secondary headaches such as epilepsy or other neurologic disorders, presence of fever ,irregular drugs usage and discontinuation of iron usage for more than one week. Topiramate as prophylactic migraine treatment started in the children based on the result of another study (4) and all of participant patients received 2mg/kg/day topiramate and 4mg/kg/day ferrous sulfate (maximum 150 mg) for 90 consecutive days. The drugs were administered orally in single dose at bedtime. 

The children were visited monthly for three consecutive months by the intern of research in Pediatric Neurology Clinic of Shahid Sadoughi Hospital and clinical information about frequency, severity and duration of headaches, Pediatric Migraine Disability Assessment score (14) and clinical side effects of the drugs were recorded. Severity of headache was assessed by asking each child to grade majority of headache pain on visual analogue scale (VAS) (15) on 10-point scale as no pain = scale of 0 and the most severe pain = 10. A VAS is a horizontal or vertical 10 cm long line which is marked at the extremes with “no pain and worst pain imaginable”. The children were asked to place a mark on the line that represented their pain level. The drugs were continued for 90 consecutive days and then, monthly frequency, severity and duration of headache and pedMIDAS before and after three months of drugs usage were compared. The data were analyzed using SPSS Version 17 statistical software. Chi-square test or Fisher exact test was used for data analysis of qualitative variables and mean values in iron deficient children before and after treatment were compared by paired t-test and mean values in the two groups ( without and with iron deficiency) were compared by independent t-test. Differences were considered significant at p value of less than 0.05. The study had been approved by the Ethics Committee of Shahid Sadoughi University of Medical Sciences, Yazd, Iran. The current research was not funded by any drug company.

## Results

In this study, 98 children, including 46 girls (46/9%) and 52 boys (53/1%), with mean age of 9/72 ± 3/19 were evaluated. Based on the interpretation of laboratory tests function, 31 children (31/6%) had iron deficiency and 10 children (10.2%) had iron deficiency anemia.Comparison of some characteristics of the children in both groups (migraineur children without and with iron deficiency) is shown in Table I .As it shows that age and onset age of migraine ,sex distribution, type of migraine and positive family history of migraine were not statistically significant different in both groups. Table II presents comparison of headache characteristics in both groups which indicates that in children with iron deficiency, monthly frequency, severity, and disability of headache were more than in children without iron deficiency ,but, duration of headache was not statistically significant different in with and without iron deficient children.Comparison of headache characteristics of migraineur children with iron deficiency before and after iron therapy is presented in Table III which shows that ferrous sulfate treatment was effective in reduction of monthly frequency, severity score, duration and disability score of headache. One child loosed to follow-up and three children discontinued iron usage. So, they were excluded. Serious clinical side effects were not observed in any of the patients, but, in 27 children who completed study period, daily sleepiness, as a side effect of topiramate was seen in two and iron therapy side effects were seen in four of them (14.8%) including nausea and vomiting in two persons, constipation in one and heartburn in one child.

**Table I:** *Comparison of some characteristics of children in the two groups*

**Variables**	**With iron deficiency**	**Without iron deficiency**	**p-value**
Age (mean ± SD)	10.11 ± 3.12	10.22 ± 2.98	0.4
Age of migraine onset (mean ± SD)	7.35 ± 2.78	8.01 ± 1.95	0.7
Sex			
Female	17	29	0.01
Male	14	38	
Type of migraine			
Without aura	20	41	0.5
With aura	11	26	
Positive family history of migrane			
Yes	26	57	0.5
No	5	10	

**Table II:** *Comparison of headache characteristics in both groups*

**V**aribles	With iron deficiency	Without iron deficiency	**p-** value
Monthly headache frequency	22.89 ± 7.18	14.5 ± 4.56	0.02
Headache duration in hours	2.14 ± 1.23	2.26 ± 1.63	0.3
Severity score of headache	8.12 ± 1.76	5.03 ± 1.15	0.02
Headache disability score: pedMIDAS	38.23 ± 10.7	30.12 ± 7.46	0.03

**Table III:** *Comparison of headache characteristics before and after iron therapy*

**Varibles**	Before treatment	After treatment	p- value
Monthly headache frequency	22.89 ± 7.18	10.13 ± 4.51	0.001
Headache duration in hours	2.14 ± 1.23	1.14 ± 1.01	0.001
Severity score of headache	8.12 ± 1.76	5.11 ± 1.62	0.001
Headache disability score	38.23 ± 10.7	22.87 ± 8.65	0.01

## Discussion

Neurologic manifestations of iron deficiency in children include developmental delay, stroke, breath-holding episodes, pseudotumor cerebri, and cranial nerve and iron deficiency identification is uncommon as part of the differential diagnosis in such disorders (16). Trace elements such as zinc, copper and iron might have a role in pathophysiology of acute migraine headache (17, 18). In addition, in women with pure menstrual migraine and menstrual related migraine iron deficiency, anemia is more common which might be an important mechanism in increasing of migraine attacks (19). In the present study, 31.6% of 5-15 years old migraineur children had iron deficiency and 10.4% had iron deficiency anemia. In Pamuk et.al study in Turkey, 36.2% of iron deficient patients had migraine headache (20), therefore it seems logical to measure serum iron and ferritin in patient with migraine.Result of this research showed that monthly frequency, severity score and disability score of headache were more in children with iron deficiency that is in compliance with another Iranian study that showed iron deficiency was more frequent in 15-45 years old women with migraine headache than in women without migraine headache (8).In the present study, 2 mg/kg/day topiramate plus 4 mg/kg/day ferrous sulfate was effective in reducing monthly frequency, duration, severity and disability of headache of children with migraine and safety and efficacy of ferrous sulfate therapy is similar to another Iranian study that 400 mg/day of sodium valproate and 150 mg/day of ferrous sulfate were effective in reducing monthly frequency and severity of migraine headache of 15-45 years old women with and without iron deficiency (9). Also, in Ghasemy et al. study in Iran, Arak, ferrous sulfate therapy for three consecutive months in fifty women in reproductive age who had iron anemia and vascular headaches was effective in reduction of headache monthly frequency and number of analgesic usage during follow up period (21). In present research, ferrous sulfate was well tolerated and no life-threatening clinical side effects were seen in children and safety of ferrous sulfate therapy was similar to another study on 59 children with iron deficiency anemia (22).

## Conclusion

Based on results of this research, iron deficiency in 5-15 years old children increased monthly frequency, severity score and disability score of migraine headache and iron therapy was effective in reducing of monthly frequency, duration, severity and disability of migraine headache. Therefore, in children with migraine, evaluation of iron status and measurement of serum iron and ferritin should be done and ferrous sulfate might be used as a safe and effective drug for prevention of migraine headaches in them.

## References

[1] Hershey AD, Kliegman RM, Stanton BF, Schor NF, S. Geme JW, Behrman RE (2011). Headaches. Nelson Textbook of Pediatrics.

[2] Bonfert M, Straube A, Schroeder AS, Reilich P, Ebinger F, Heinen F (2013). Primary headache in children and adolescents: update on pharmacotherapy of migraine and tension-type headache. Neuropediatrics.

[3] Victor S, Ryan S (2014). Drugs for preventing migraine headaches in children. Cochrane Database Syst Rev.

[4] Fallah R, Divanizadeh MS, Karimi M, Mirouliaei M, Shamszadeh A (2013). Topiramate and propranolol for prophylaxis of migraine. Indian J Pediatr.

[5] Yadav D, Chandra J (2011). Iron deficiency: beyond anemia. Indian J Pediatr.

[6] Mirmiran P, Golzarand M, Serra-Majem L, Azizi F (2012). Iron, iodine and vitamin A in the Middle East; a systematic review of deficiency and food fortification. Iran J Public Health.

[7] Keikhaei B, Zandian K, Ghasemi A, Tabibi R (2007). Iron-deficiency anemia among children in southwest Iran. Food Nutr Bull.

[8] Keivani Z, Mirzaei M, Mahmoudzadeh M, Etemadifar SH, Rafieian M (2010). Evaluation the relationship between iron deficiency anemia and migraine headache in patients who referred to neurology clinic of Shahrekord University of Medical Science. Iran J Nurs Res.

[9] Gholamreza-Mirzaee M, Kheiri S, Khosravi Sh, Koshdel A, Keyvani Z, Amini Z (2012). Iron therapy and migraine headache. J Shahrekord Univ Med Sci.

[10] Oleson J (2004). The International Classification of Headache Disorders: 2nd edition. Headache Classification Sub committee of the International Headache Society. Cephalalgia.

[11] Carvalho AG, Lira PI, Barros Mde F, Aléssio ML, Lima Mde C, Carbonneau MA, Berger J, Léger CL (2010). Diagnosis of iron deficiency anemia in children of Northeast Brazil. Rev Saude Publica.

[12] Van Vranken M (2010). Evaluation of microcytosis. Am Fam Physician.

[13] Idro R, Gwer S, Williams TN, Otieno T, Uyoga S, Fegan G (2010). Iron deficiency and acute seizures: results from children living in rural Kenya and a meta-analysis. PLoS One.

[14] Hershey AD, Powers SW, Vockell ALB, LeCates SL, Kabbouche MA, Maynard MK (2001). PedMIDAS: Development of a questionnaire to assess disability of migraines in children. Neurology.

[15] Wewers ME, Lowe NK (1990). A critical review of visual analogue scales in the measurement of clinical phenomena. Res Nurs Health.

[16] Yagar JY, Hartfield DS (2002). Neurologic manifestation of iron deficiency anemia in childhood. Pediatr Neurol.

[17] Gonullu H, Gonullu E, Karadas S, Arslan M, Kalemci O, Aycan A, Sayin R, Demir H (2015). The levels of trace elements and heavy metals in patients with acute migraine headache. J Pak Med Assoc.

[18] Dhillon KS, Singh J, Lyall JS (2011). A new horizon into the pathobiology, etiology and treatment of migraine. Med Hypotheses.

[19] Vuković-Cvetković V, Plavec D, Lovrencić-Huzjan A, Galinović I, Serić V, Demarin V (2010). Is iron deficiency anemia related to menstrual migraine? Post hoc analysis of an observational study evaluating clinical characteristics of patients with menstrual migraine. Acta Clin Croat.

[20] Pamuk GE, Top MŞ, Uyanık MŞ, Köker H, Akker M, Ak R, Yürekli ÖA, Çelik Y (2015). Is iron-deficiency anemia associated with migraine? Is there a role for anxiety and depression?. Wien Klin Wochenschr.

[21] Ghasemy K, Asghari A, Narenji F, Moshfeghi K, Eshrati B (2010). The effect of using iron tablet on decreasing vascular headache in women at productive age. J Arak Univ Med Sci.

[22] Bopche AV, Dwivedi R, Mishra R, Patel GS (2009). Ferrous sulfate versus iron polymaltose complex for treatment of iron deficiency anemia in children. Indian Pediatr.

